# Comparison of CO_2_ Vertical Profiles in the Lower Troposphere between 1.6 µm Differential Absorption Lidar and Aircraft Measurements Over Tsukuba

**DOI:** 10.3390/s18114064

**Published:** 2018-11-21

**Authors:** Yasukuni Shibata, Chikao Nagasawa, Makoto Abo, Makoto Inoue, Isamu Morino, Osamu Uchino

**Affiliations:** 1 Faculty of System Design, Tokyo Metropolitan University, Tokyo 1910065, Japan; nagasawa@tmu.ac.jp (C.N.); abo@tmu.ac.jp (M.A.); 2 Department of Biological Environment, Akita Prefectural University, Akita 0100195, Japan; makoto@akita-pu.ac.jp; 3 National Institute for Environmental Studies, Ibaraki 3058506, Japan; morino@nies.go.jp (I.M.); uchino.osamu@nies.go.jp (O.U.)

**Keywords:** differential absorption Lidar, CO_2_, aircraft, vertical profile

## Abstract

A 1.6 μm differential absorption Lidar (DIAL) system for measurement of vertical CO_2_ mixing ratio profiles has been developed. A comparison of CO_2_ vertical profiles measured by the DIAL system and an aircraft in situ sensor in January 2014 over the National Institute for Environmental Studies (NIES) in Tsukuba, Japan, is presented. The DIAL measurement was obtained at an altitude range of between 1.56 and 3.60 km with a vertical resolution of 236 m (below 3 km) and 590 m (above 3 km) at an average error of 1.93 ppm. An in situ sensor for cavity ring-down spectroscopy of CO_2_ was installed in an aircraft. CO_2_ mixing ratio measured by DIAL and the aircraft sensor ranged from 398.73 to 401.36 ppm and from 399.08 to 401.83 ppm, respectively, with an average difference of −0.94 ± 1.91 ppm below 3 km and −0.70 ± 1.98 ppm above 3 km between the two measurements.

## 1. Introduction

Before the Industrial Revolution, the atmospheric levels of carbon dioxide (CO_2_) were around 280 ppm [1]. On May 9 2013, the daily average concentration of CO_2_ in the atmosphere surpassed 400 ppm for the first time at the Mauna Loa Observatory in Hawaii, where the modern record of observations began back in 1958 [2]. IPCC 2013 has reported that the concentration will reach at least 440 ppm—more than 1.5 times the preindustrial level—by 2050 [1].

Highly accurate vertical CO_2_ profiles are desirable to improve quantification and understanding of the global sink and source of CO_2_ and of global climate change [3]. Validating and improving the global atmospheric transport model requires precise measurement of CO_2_ profile. Atmospheric CO_2_ concentrations have been measured with high accuracy at ground stations and tall towers as well as on ships, aircraft, and balloons using flask sampling or continuous measurement equipment. In comparison with the ground-based measurements, measurements of CO_2_ vertical profiles in the troposphere have been limited as the measurements conducted using campaign-style aircrafts and commercial airlines have limited spatial and temporal coverage [4,5,6,7,8].

Light detection and ranging (Lidar) is one of the best methods for observing the vertical distribution of greenhouse gases. The differential absorption Lidar (DIAL) method with its high range resolution is expected to bring several advantages over passive measurements, for example, daytime and nighttime coverage and negligible influences of aerosol and cirrus layers [9,10,11]. DIAL operates at two wavelengths, one on resonance and one off resonance of the molecular absorption of the gas of interest. Because the on resonance wavelength is more strongly absorbed by the gas, measurement of the ratio of the backscatter at the two wavelengths as a function of range can be used to calculate the gas concentration profile. Many CO_2_ absorption bands exist between 0.7 and 10 µm wavelengths, and each band contains many absorption lines. Two bands, particularly 1.6 and 2.0 μm, are suitable for DIAL measurements and the 1.6 μm band is the very interesting. It avoids contamination from other atmospheric constituents such as water vapor. Furthermore, the peak absorption intensity of the 1.6 μm absorption spectrum is suitable for DIAL measurements of vertical profiles in the troposphere. Some studies have suggested using the 2.0 μm band where an absorption peak line, they are limited to measure the distribution or the column amount in the range of several hundred meters due to strong absorption [12,13,14,15]. Moreover, although some studies have suggested using the 2.0 μm band where an off-center absorption line is usually possible to have an adaptive absorption for measurements in the troposphere [16,17], a 1.6 μm system benefits from a relatively lower magnitude of the absorption cross section at the center absorption line. Using 1.6 μm DIAL systems, the system error due to the fluctuation of the laser frequency is small, because tuning the laser wavelength to the center absorption line is easier than tuning to the off-center absorption line.

We have developed a 1.6 μm optical parametric generator (OPG)/optical parametric amplifier (OPA) transmitter and used it in a direct-detection DIAL system to measure CO_2_ mixing ratio profiles [18]. The 1.6 μm OPG/OPA transmitter system for the CO_2_ DIAL system is pumped by an iodine-based Q-switched Nd:YAG laser with a 500 Hz repetition rate. The optical receiver includes a near-infrared photomultiplier tube (PMT) with high quantum efficiency operating in the photon counting mode and a narrowband interference filter with a 1.0 nm full width at half maximum (FWHM) for daytime measurements. We conducted a field experiment at the Hino campus of Tokyo Metropolitan University to compare CO_2_ DIAL measurements with surface in situ sensor measurements to validate the DIAL measurements. An open-path CO_2_ gas analyzer (LICOR. Inc., LI-7500, Lincoln, NE, USA) is installed at the top of building at a height of 42 m. The CO_2_ DIAL is installed on the first floor of another building 110 m away from that building. The laser beam is irradiated directly over the gas analyzer. Data from the CO_2_ DIAL are acquired with a range resolution of 60 m and an integration time of five minutes. Data on the CO_2_ mixing ratio of the gas analyzer are acquired every one second. We found the difference between the CO_2_ mixing ratio measurements to be 0.06 ppm at 10 min average intervals [18]. As a next step, we conducted a field experiment to compare CO_2_ DIAL measurements with in situ sensor measurements on an aircraft to validate the vertical DIAL measurements. In this paper, we report a comparison of measurements of vertical distributions of CO_2_ mixing ratio by DIAL and the aircraft sensor.

## 2. Experimental Setup

### 2.1. CO_2_ DIAL

**Figure 1:**
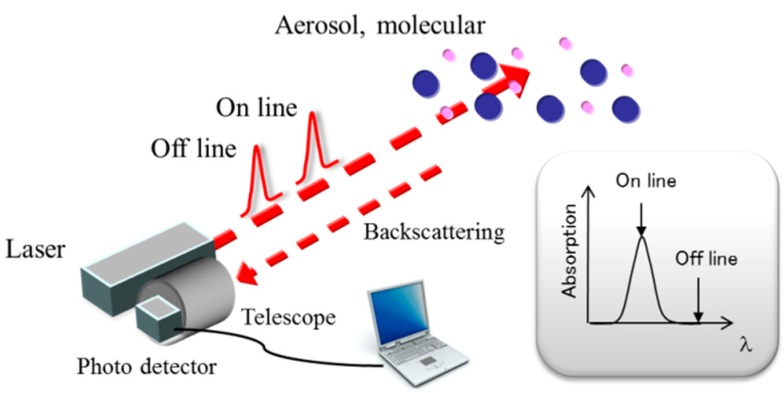
Schematic illustration of the differential absorption Lidar (DIAL) system.

Figure 1 shows a schematic illustration of the DIAL system. The DIAL technique uses the absorption properties of a target gas to deduce its atmospheric concentration. Laser beams at two different wavelengths are sent into the atmosphere. The wavelengths are chosen such that one of them is absorbed more (on-line wavelength, λ_on_) than the other (off-line wavelength, λ_off_). The difference in the absorption along the beam path causes the returned Lidar signals to yield different range dependence. The average gas density *n* [/m^3^] between ranges *R*_1_ and *R*_2_ is given by the DIAL equation [19].

(1) $$n=\frac{1}{2\Delta \sigma \left|R_{1}-R_{2}\right|}\ln\left[\frac{S_{on}\left(R_{1}\right)S_{off}\left(R_{2}\right)}{S_{on}\left(R_{2}\right)S_{off}\left(R_{1}\right)}\right]$$

where *S*_on_ and *S*_off_ are the Lidar signals at λ_on_ and λ_off_, respectively, and Δσ is the differential absorption cross-section between λ_on_ and λ_off_. The mixing ratio is the ratio of the air density and the gas density 𝑛. Because atmospheric temperature and pressure are related by the ideal gas law, the mixing ratio is also related by the atmospheric temperature and pressure. The random error in the measurement of a signal *S* is calculated from Poisson statistics as √*S*. The relative error in n for a DIAL measurement in the photon counting mode is given by

(2) $$\frac{\Delta n}{n}=\frac{1}{2n\Delta \sigma \left|R_{1}-R_{2}\right|}\sqrt{\sum_{i=1}^{2}\sum_{j=1}^{2}\frac{S_{ij}+B}{S_{ij}^{2}}},$$

where *i* = 1, 2 denote ranges *R*_1_ and *R*_2_, respectively, and *j* = 1, 2 denote the on-line and off-line signals, respectively. *B* is the background noise.

**Figure 2:**
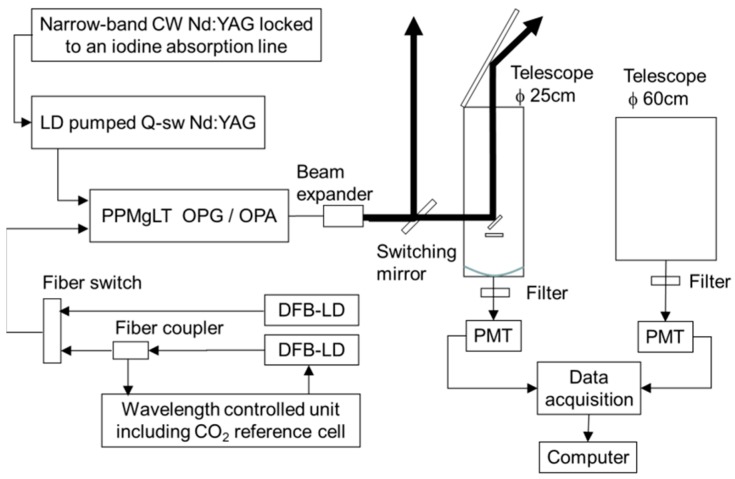
Block diagram of the 1.6 µm DIAL system for measurement of CO_2_ mixing ratio profiles. The low-altitude mode measurements were performed using a 25-cm-diameter telescope and the high-altitude mode measurements were done using a 60-cm-diameter telescope.

Figure 2 shows a block diagram of the 1.6 μm CO_2_ DIAL system for comparison of measurements of vertical profiles of CO_2_ dry mole fractions by DIAL and the aircraft sensor. The system parameters are summarized in Table 1. To stabilize the oscillation wavelength of the OPG, an iodine locked seed laser was used for the pulsed Nd:YAG laser. The OPG output with an injection seeder was amplified by the OPA. The partial power of the on-line injection seeder (distributed feedback laser) was split off and directed through a wavelength-controlled unit. The on-line wavelength (1572.992 nm) laser was tuned to the absorption line center and stabilized by a feedback control unit using a CO_2_ absorption cell. The off-line wavelength (1573.137 nm) laser was operated in the free-run mode. Both the on-line and off-line distributed feedback (DFB) lasers were connected to an optical fiber switch and the switching speed was 250 Hz. The wavelength stability is measured by a wavemeter (HighFiness WS7/IR, Tübingen, Germany) within a 10 MHz frequency resolution for 2 h. It is estimated that the expected fluctuation in the wavelength is suppressed by less than 10 MHz. The DIAL measurement errors associated with a laser frequency uncertainty of <10 MHz are calculated to be 0.1%. The injection-seeded OPG generates a strong narrow signal and a broad side lobe with a 1.4 nm spectral width. The line width of the strong signal is less than 280 MHz. This side lobe results from a non-collinear phase-matched process, which experiences a significant overlap with the large pump beam. The measurement error by using the absorption cross section when compensating for the broad side lobe can be calibrated [18].

The dynamic range limitation of the receiving system made it difficult to measure from near the ground to an altitude of 5 km. Therefore, the two receiving systems were prepared: a “low-altitude” mode to target an altitude lower than 2.5 km and a “high-altitude” mode to target an altitude higher than 2.5 km. The atmospheric backscatters were collected by a 250 mm Schmidt–Cassegrain telescope with a field-of-view (FOV) of 1 mrad for the low-altitude mode and a 600 mm Schmidt–Cassegrain telescope with a FOV of 1 mrad for the high-altitude mode. The pulsed laser output was transmitted vertically into the atmosphere by a switching mirror for both low-altitude and high-altitude measurements. The collected scattered light was sent to the near-infrared PMT module (Hamamatsu Photonics K.K. H10330A-75, Hamamatsu, Japan) operated in the photon-counting mode. This PMT is contained in a thermally insulated sealed-off housing evacuated to a high vacuum. The internal thermoelectric cooler eliminates the need for liquid nitrogen and cooling water. The quantum efficiencies of the low-altitude and high-altitude modes were 2% and 8%, respectively. Because the backscattering light was strong for the low-altitude measurement, we attached an appropriate neutral density (ND) filter. We performed the CO_2_ mixing ratio measurement for daytime by using the 1.6 μm DIAL with a 1.0-nm-FWHM narrowband interference filter and a PMT.

### 2.2. Aircraft

Beechcraft King Air 200T, operated by Diamond Air Service Inc.,Toyoyama, Japan is a twin-turboprop aircraft with a pressurized cabin. We installed an in situ sensor with a nondispersive infrared gas analyzer (NDIR; LICOR. Inc., LI-840, Lincoln, NE, USA) and a cavity ring-down spectrometer (CRDS; Picarro, G2301-m, Santa Clara, CA, USA) for CO_2_ and methane (CH_4_). CRDS measurements are rapid and highly sensitive for measuring CO_2_ and CH_4_ mixing ratio [20]. A pulsed beam from a single-frequency laser diode enters an optical cell with highly reflective mirrors (typically R > 99.9%). The light transmitted through the exit mirror is measured with respect to time. Under certain conditions, the resulting signal decays exponentially with time. The decay time depends on the reflectivity of the two mirrors, the distance between the two mirrors, the speed of light, and the molecular absorption coefficient of absorbing species in the cavity. CO_2_ and CH_4_ mixing ratio are derived from absorption at selected spectral lines every 2 seconds. The time delay caused by the distance between the inlet and the CRDS is corrected. The CRDS is calibrated with standard gas before the flight. CRDS measurements were performed under moderate dehumidification of air samples. In our study, simultaneous H_2_O measurements were performed to correct these mixing ratio to dry mole fractions by the CRDS. We corrected the CRDS data using the following equation shown by Nara et al. [21].

(3) $$X_{\mathrm{dry}}=\frac{X_{\mathrm{wet}}}{1-a{\left[H_{2}O\right]}_{\mathrm{CRDS}}-b{\left[H_{2}O\right]}_{\mathrm{CRDS}}^{2}}$$

where *X*_dry_ is CO_2_ or CH_4_ mixing ratio corrected by the water correction function, and *X*_wet_ is observation by the CRDS. [*H_2_O*]_CRDS_ indicates water vapor concentration reported by the CRDS. Estimated linear *a* and quadratic *b* terms of CO_2_ are 0.01204 and 0.00025. Estimated linear *a* and quadratic *b* terms of CH_4_ are 0.00999 and 0.00014.

We also performed flask sampling at eight altitude levels to check accuracy of the in situ CO_2_ profile and to obtain mixing ratio of other trace gases such as CH_4_, CO, N_2_O, H_2_, and SF_6_ [22]. Typical durations of spiral descent flights were about 1 h between 33,000 ft (9900 m) and 1600 ft (480 m). Vertical profiles of pressure, temperature, relative humidity, wind direction, and wind speed were monitored by the aircraft’s instruments.

## 3. CO_2_ DIAL and Aircraft Campaign

**Figure 3:**
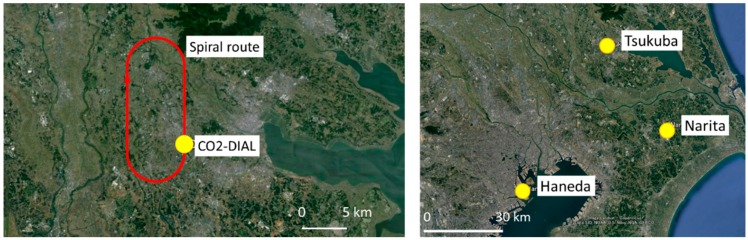
Observation sites where CO_2_ DIAL (**right**) and aircraft measurements (**left**) were made on 12 January 2014 over Tsukuba.

**Figure 4:**
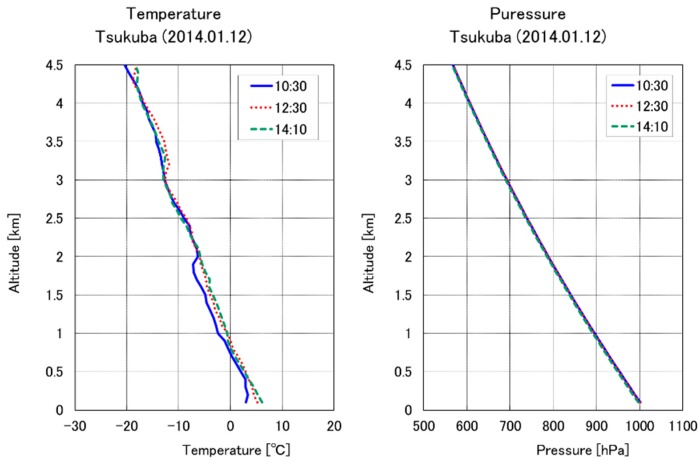
GPS sonde temperature (**left**) and pressure (**right**) profiles launched from Tsukuba on 12 January 2014.

In January 2014, two aircraft campaigns were made above the National Institute for Environmental Studies (NIES) in Tsukuba, Japan. Figure 3 shows the location of the observation site. Tsukuba is 50 km northeast of Tokyo and includes forests, agricultural lands, and urban areas. The aircraft took off from Sendai Airport, Miyagi, Japan. The CO_2_ DIAL system was installed in a mobile container for measurements of CO_2_ mixing ratio profile at the NIES campus. The size of the mobile container is 6096 L × 2438 W × 2621 H (mm), and it is towed by other cars. 200 VAC power supply for the Nd:YAG laser and 100 VAC are supplied externally. The room temperature of the container is controlled by the air conditioner. The OPG/OPA transmitter system is housed in a cover with air purifier and air conditioner to avoid dust. CO_2_ DIAL and aircraft campaign helds on 12 January 2014 at the NIES site. Because of the air traffic control restrictions for the controlled airspace of the Narita and Haneda international airports, the lowest flight altitude of the aircraft was 480 m over Tsukuba. Additional vertical profiles of pressure, temperature, relative humidity, wind direction, and wind speed were obtained by GPS sondes at NIES, Tsukuba, Japan. Figure 4 shows temperature and pressure profiles obtained by the GPS sondes for 12 January 2014. Temperature resolution is 0.1 °C and pressure resolution is 0.1 hPa. GPS sondes were launched at 10:30 LT, 12:30 LT, and 14:10 LT. Ground-based CO_2_ measurements were simultaneously performed using a NDIR at the Meteorological Research Institute, Tsukuba, Japan.

**Figure 5:**
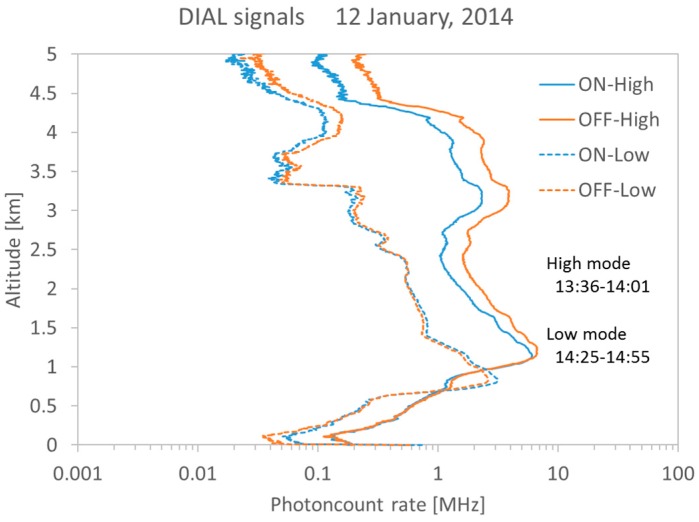
On-line and off-line return signals with high-altitude mode and low-altitude mode. The range resolution is 7.5 m.

**Figure 6:**
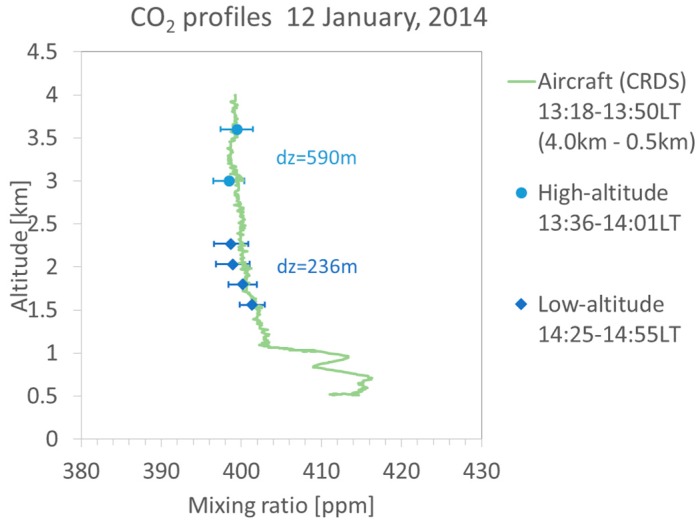
Comparison of CO_2_ vertical mixing ratio profiles observed by DIAL and aircraft. dz: vertical resolution.

The CO_2_ DIAL system obtained the CO_2_ vertical mixing ratio profile for 1.5 to 3.5 km altitude from 13:36 to 14:55 LT on 12 January 2014. Figure 5 shows on-line and off-line return signals. The PMT signals are digitized by a LICEL transient recorder TR20-80 with 12 bit resolution, 20 MHz sampling rate equivalent to 7.5 m range resolution. Overlap altitudes are 0.8 km in the low-altitude mode and 1.1 km in the high-altitude mode. Rich aerosol backscattered signal is detected between 3 and 4 km altitude in the high-altitude mode, and is detected below 3.2 km altitude in the low-altitude mode. Figure 6 shows the comparison of DIAL data and aircraft (CRDS) data profiles (13:18 to 13:50 LT). The CRDS CO_2_ data were obtained from low altitude (0.5 km) to upper atmosphere (4.0 km). DIAL measurements were performed in the high-altitude mode from 13:36 to 14:01 LT and in the low-altitude mode from 14:25 to 14:55 LT. The optical density of the ND filter used in the low-altitude mode was 0.7. Vertical resolutions of the CO_2_ DIAL system were 236 m below 3 km and 590 m above 3 km. CO_2_ density obtained by DIAL measurement is calculated from Equation (1) and CO_2_ mixing ratio is obtained by air density calculated from temperature and pressure by a GPS sonde launched at 14:10 LT. The relative error of the DIAL data were calculated by Equation (2) and is shown as error bars in Figure 6. Table 2 shows the differences between CO_2_ mixing ratio derived from CO_2_ DIAL and aircraft measurements at various altitudes. The vertical resolution of CRDS measurements are adjusted to the CO_2_ DIAL measurements. In the low-altitude mode, the average relative error of the CO_2_ DIAL system was 1.91 ppm and the average difference between the values observed by the CO_2_ DIAL system and the aircraft sensor was −0.94 ± 1.91 ppm. In the high-altitude mode, the average relative error of the CO_2_ DIAL system was 1.98 ppm and the average difference between the values observed by the CO_2_ DIAL system and the aircraft sensor was −0.70 ± 1.98 ppm. The CO_2_ mixing ratio reported by aircraft observations were almost within the error bar of DIAL observations. The CO_2_ DIAL system is, therefore, capable of performing highly accurate vertical CO_2_ mixing ratio measurements.

## 4. Conclusions

We present the first evaluation of a campaign for measurement of CO_2_ mixing ratio vertical profiles with the CO_2_ DIAL system and a CRDS sensor onboard an aircraft over the site of NIES, Tsukuba, Japan, on 12 January 2014. CO_2_ DIAL data and aircraft CRDS data profiles were observed at 1.5 to 3.5 km altitude (13:36 to 14:55 LT) and 0.5–4.0 km altitude (13:18 to 13:50 LT). The CO_2_ mixing ratio profiles show excellent consistency within the error bars of the CO_2_ DIAL system, and the average difference between the CO_2_ DIAL and aircraft sensor measurements was −0.94 ± 1.91 ppm below 3 km and −0.70 ± 1.98 ppm above 3 km. These results demonstrate that the 1.6 μm direct-detection CO_2_ DIAL system can measure vertical CO_2_ mixing ratio profiles with high accuracy in the lower troposphere. Measurements of vertical CO_2_ profiles using the CO_2_ DIAL system can contribute to understanding forest CO_2_ flux without using towers and contribute to understanding CO_2_ flux from industrial and suburban areas.

## Figures and Tables

**Table 1:** Parameters of the 1.6 µm DIAL system.

	Low-Altitude	High-Altitude
Pulse Energy	6 mJ	
Laser Wavelength	On: 1572.992 nm, Off: 1573.137 nm	
Telescope Diameter	25 cm	60 cm
Interference Filter	1.0 nm FWHM	
Quantum Efficiency	2 %	8 %
Detection Scheme	Photon counting mode	

**Table 2:** Comparison of CO_2_ mixing ratio derived from CO_2_ DIAL and aircraft measurements. (*Difference: DIAL − aircraft).

Altitude [m]	Vertical Resolution [m]	CO_2_ DIAL [ppm]	Relative Error (CO_2_ DIAL) [ppm]	Aircraft (CRDS) [ppm]	*Difference [ppm]
1560	236 (low-altitude)	401.36	1.59	401.83	−0.47
1797		400.16	1.79	400.68	−0.51
2033		398.97	2.10	400.47	−1.50
2269		398.73	2.16	400.01	−1.28
3002	590 (high-altitude)	398.43	1.93	399.50	−1.07
3599		399.44	2.03	399.08	0.37
